# Diagnostic reproducibility on whole digital slide in cytology and histology in oncologic screenings

**DOI:** 10.1186/1746-1596-8-S1-S12

**Published:** 2013-09-30

**Authors:** Stefania Lega, Paola Crucitti, Paola Pierotti, Roberta Rapezzi, Priscilla Sassoli de’ Bianchi, Carlo Naldoni, Arrigo Bondi

**Affiliations:** 1 Anatomia Patologica Ospedale Maggiore, Azienda USL di Bologna, Italy; 2 Sanita’ e Politiche Sociali – Regione Emilia-Romagna, Italy

## Background

Diagnostic reproducibility and accuracy in cytology and histology are major issues in Oncologic Screenings of cervix, breast and colorectal cancer : it can be achieved by procedures and programs for quality assurance (QA). The slides set standard represents the most used method to compare diagnostic proficiency, the chance of interpreting microscopic digital photographs provided an interesting alternative to reading conventional microscope slides.

The whole digital slide observed in a computer screen is a third, interesting, option to reach the purpose. In fact all the information on conventional sample are transferred into a file, easily archived, cataloged, duplicated or advice for quality control, but is especially available at a distance and from multiple locations simultaneously with drastic reduction of time needed to achieve proficiency test reproducibility [1].

The production of digital slides with modern scanners is relatively simple and quick. All suppliers offer publishing services into private or public networks server [2] and software able to track scanned cases comprehensive database to build large casistic archives on the net [3].While tools are already available for a teleconference discussion of cases with vision of cytological preparations on line [4,5]**,** educational programs with integrated digital slides are poorly developed nor self-evaluation or proficiency tests for continuing education and professional updating are easily accessible.

A project on Virtual Microscopy and Digital Pathology has been conducted in Emilia-Romagna (Italy) with the objective to promote quality in diagnostic cytology and histology of Screenings by testing a different system involving pathologists and cytologists using digital slides, with a faster mechanism and reproducible than standard diagnostic sets and by distance training operators with a final consensus diagnosis meeting.

The aim has been reached with the realization of a management system for cytological and histological whole-slides digital images and related clinical data and the building of a picture archive and communication system (PACS) among pathologists of our Region. This must be backed by software for the realization of network slide seminars to perform periodic tests of diagnostic reproducibility and proficiency test. The cases, collected and properly cataloged in an online, extensive and systematic digital archive of slides, easily accessible, with diagnoses discussed in clinical-pathology audit and validated by experts, can be used as diagnostic reference tool (casistic Atlas online ). The cataloging and indexing is performed with NAP codes, a Nomenclature derived from SNOMED [6], which contains terms in Italian and English and encompasses extensive synonyms and complex searches.

## Material and methods

**Figure 1:**
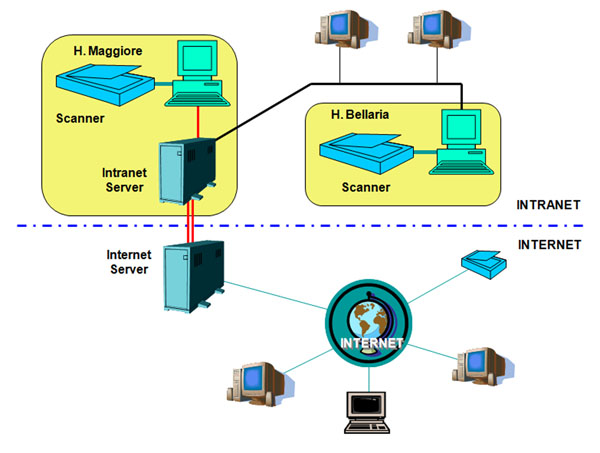
Network scheme: two scanner are in an intranet environment with an local disk server, connected to an internet server where the public products are stored.

The cancer screening survey group of the Emilia Romagna Region (Italy) set up a picture, archive and communication system (PACS) devoted to pathologists for cooperative diagnosis, didactics and training, teleconsulting, documentation of rare cases and pilot experiences; furthermore selected cases are catalogued in the PACS with the aim of the check of the diagnostic concordance in the regional oncologic screenings (cervix, breast and colon). The PACS system is composed by two Aperio scanner and an adequate internet server where the described programs operate (see Figure 1) [7].

The slides have been digitalized using an Aperio scanner, 20x for histology and 40x for cytology and an internet server was used to store the files, arranged into a Spectrum database (Aperio). An e-learning platform (Docebo)[8] has been used to built an interface for the applicants: cases and slides were considered “teaching objects” for the educational software (Seminars) and appropriate questioning forms have been designed with the diagnostic occurrences of the Bethesda System 2011 for cytology and the CIN options for histology for the cervical cancer and of International Guidelines for breast and colorectal cancer.

At the present the diagnostic reproducibility has been performed in colorectal and cervical cancer screening (Bologna October 2010, Bologna June 2011), and for breast cancer is ongoing (Bologna June 2012). In all three Seminars a number of cases have been selected by a committee of Pathologists from Regional Units.

Colorectal cancer screening was certainly the first courses for pathologists performed with these features in our region and maybe in Italy.

Three Regional Units (Bologna, Cesena and Ferrara) were involved by sending representative histological cases of all main diagnostic occurrences to test the diagnostic reproducibility, 28 histological cases were represented. A day interval was let to study slides, then a consensus conference has been programmed in the same day.

In cervical cancer screening, the second Seminar of QA, to test the diagnostic reproducibility, 30 cytological and 30 histological cases have been selected by a committee of Pathologists among the cases proposed by all the Regional Units. All main diagnostic occurrences were represented, basic clinical information and relevant follow up information were available; the cases have been completely anonymized for the participant.

A 30 days interval was let to study the slides, then a consensus conference has been programmed. Before the meeting each participant received a report with the interpretation of the committee and her/his diagnosis inserted in the form.

## Results and discussion

15 Pathologists of Regional Units attended the **colon-rectal QA** and the diagnostic reproducibility have been evaluated matching them with the final diagnosis reached during the consensus conference. The observed agreement was 69% and the overall performance of the participating readers was assessed with a statistical analysis using Cohen’s kappa: the average value was 0.64 (substantial).

95 cytologists and 32 histopathologists have been involved in the **cervical cancer screening QA**.

The diagnostic reproducibility has been evaluated using the final diagnosis reached in the Consensus Conference: in 2 out of 30 cytological cases the diagnosis was different from the opinion of the committee, while all histology diagnoses were in agreement. The overall performance of the participating readers is reported in table 1.

**Table 1:** Screening PAP test - Distribution of readers' diagnosis

		Agreement in Omogeneous groups of diagnoses			
		neg	ASCUS L SIL	ASC-H H SIL / Ca sq	AGC AIS / ADK
**readers' diagnosis**	**neg**	**85,1**	15	6,6	2,1
	
	**ASCUS/ L SIL**	5,1	**72,4**	20,2	3,6
	
	**ASC-H/ H SIL/ Ca sq**	2,9	11,9	**67,7**	14
	
	**AGC / AIS/ ADK**	2,9	0,3	5,2	**79,2**
	
	**advanced tumor**	3,5	0,2	0,2	0,7
	
	**no answer**	0,5	0,2	0,1	0,4

		**100%**	**100%**	**100%**	**100%**
					
	Observed agreement		73%		
	Cohen's Kappa		0.63	(substantial)	

## Conclusion

Whole digital slide is suitable for proficiency tests and the internet e-learning platform allow to share cases and to get the answers from participants, in a easier way than in the circulation of a set of conventional slides.

The quality of whole slides is diagnostic, approaching optical microscopic resolution.

In a cytological environment the difficulty to get a perfect focus on all fields, the wider area of slide to examine and the higher number of diagnostic classes may justify a worse agreement of the users and a poorer performance (lower Cohen’s kappa) than histology.

We have produced an integrated environment that includes many of the modern aspect of digital pathology that can be shared with the PACS system in many laboratories of the Region, including quality promotion and control of image interpretation in cytology and histology applied to cancer prevention screenings.

## Competing interests

Authors declare that they have no competing interests.
